# Translation, Cross-Cultural Adaptation, and Validation of the Galician Version of the Nurse Prescribing Self-Efficacy Scale

**DOI:** 10.3390/healthcare10122563

**Published:** 2022-12-17

**Authors:** Andrea Franco-Rodríguez, Eva María Domínguez-Martís, Diego Gabriel Mosteiro-Miguéns, David López-Ares, Belén Cotón-Sánchez, Marta Domínguez-Varela, Silvia Novío

**Affiliations:** 1 Galician Public Healthcare Service, Galicia, Spain; 2 Galician Public Healthcare Service, Healthcare Centre of Concepción Arenal, C/Santiago León de Caracas 12, 15701 A Coruña, Spain; 3 Galician Public Healthcare Service, University Hospital Complex of A Coruña (CHUAC), 15006 A Coruña, Spain; 4 HM La Esperanza, Avda das Burgas 2, 15705 A Coruña, Spain; 5 Pac Casa del Mar, Healthcare Centre, Avenida Ejército 2A, 15006 A Coruña, Spain; 6 Department of Psiquiatry, Radiology, Public Health, Nursing and Medicine, University of Santiago de Compostela, C/ San Francisco s/n, 15782 A Coruña, Spain

**Keywords:** nurse prescribing, psychometric properties, reliability, self-efficacy, validity

## Abstract

Low self-efficacy has been identified as one of the factors that could hinder the prescribing competence of nurses. No valid and reliable existing instruments assess Galician nurses’ confidence to prescribe. The aim of this study was to determine the reliability and validity of the Galician version of the Nurse Prescribing Self-Efficacy Scale (G-NP-SES, 19 items). The study was conducted in two phases: the translation and adaptation to the Galician version of the NP-SES, and the assessment of its psychometric properties. G-NP-SES was sent to nurses working in primary healthcare centers in Galicia (Spain) between March and June of 2022. Its content, construct and discriminant validity, and internal consistency reliability were examined. A total of 193 people participated in the study. As the original scale, G-NP-SES also had three dimensions (% of cumulative variance = 80.82%). It showed good internal consistency (Cronbach’s alpha coefficient (α) = 0.90, with each factor ranging from 0.86 to 0.89), high content validity (scale’s content validity index = 0.92, with item-content validity index ranged from 0.87 to 1), and good discriminant validity. G-NP-SES is an instrument with good psychometric properties which could be used to accurately assess Galician nurses’ self-efficacy to prescribe and consequently to improve their job performance.

## 1. Introduction

Nurse prescribing (NP) is the process in which nurses collect information and make decisions based on their clinical judgment in order to initiate, continue, or cease treatments to meet the health needs of the population [1]. NP has a lot of advantages for the healthcare system [2], for the patients [3], and even for the nurses [4]; however, obtaining prescribing rights has not been an easy process for nurses [5].

The barriers that have hindered the prescribing competence of nurses include both those unrelated to nursing professionals and those dependent on them. The former encompasses, among other things, gender-associated constraints [5]—as well as the opposition of physicians, since it might compromise patient safety [6]. No less important are the barriers specific to nurses, such as nurses’ uncertainty about their educational preparation [7], or their lack of self-efficacy to perform NP [8]. These latter barriers explain why sometimes nurses have refused to use their legal prescribing rights on the work floor [9].

Self-efficacy, a major component of Bandura’s social cognitive theory, is the belief in one’s capacity to perform assigned functions and duties [10]. Self-efficacy has been identified as a predictor of the achievement of goals [11]. Its tight positive relationship with job motivation helps us to understand why people with a high level of professional self-efficacy face difficulties more effectively, set their own goals, and are more persistent in achieving proposed objectives than people with low self-efficacy [10,12]. In contrast, people with low self-efficacy perceive that they do not control workplaces effectively, they also tend to be less engaged and suffer more from burnouts [13]. Following this, assessment of nurses’ self-efficacy in acquisition of new competences, such as NP, is necessary because of its positive influence on patient care.

Several scales have been used to evaluate the self-efficacy of nurses [14,15,16]; however, to the best of our knowledge, the Nurse Prescribing Self-Efficacy Scale (NP-SES), developed by Galiana–Camacho et al. [8], is the only reliable and valid instrument for the assessment of nurses’ self-efficacy to prescribe. NP-SES comprises three dimensions which are in line with international competency frameworks for prescribers and are considered central to NP: clinical assessment and pharmacological knowledge, supplementary prescribing and evidence-based practice, and independent prescribing and patient education. The assessment of the level of confidence that nurses have about their pharmacological knowledge, diagnostic ability, and clinical decision-making capacity allows us to determine their self-efficacy for all the main actions of the NP process [8,17].

NP-SES has been shown to have good psychometric properties for Spanish-speaking nurses. Galicia is an autonomous region of Spain with two official languages, Spanish and Galician, where, according to the most current statistics, nearly three-quarters of the population speaks in Galician (30.57% always speaks in Galician, 21.72% speaks more often in Galician than Spanish, and 23.32% speaks more often in Spanish than Galician) [18]. Based on the above considerations, the aim of this study was to assess the reliability and validity of the Galician version of the NP-SES (G-NP-SES) in order to examine the Galician nurses’ self-efficacy to prescribe.

## 2. Materials and Methods

### 2.1. Study Design

This study was conducted in two phases. In the first phase, Galician translation and adaptation of the scale was completed. This was followed by pilot testing of the instrument. In the second phase, a cross-sectional survey was conducted in order to assess the scale’s final psychometric properties.

### 2.2. Subjects and Setting

The study population consisted of nursing professionals working in Galician primary public healthcare centers. The investigation included nurses, permanent or temporary, of either sex and of 18 years of age or older, who voluntarily agreed to participate. Participants were recruited through printed and electronic advertisements sent to primary public healthcare centers of Galicia.

For the validity and reliability studies, according to the rule of thumb from Nunnally [19], there should be at least 10 times as many subjects as items. In this study, considering the number of items in the scale (*n* = 19), the survey aimed to reach 190 nurses. 

### 2.3. Data Collection

#### 2.3.1. General Demographic Data

A self-designed form about the participants’ sociodemographic information included age, sex, education, occupation, and years of work experience of the nurses. According to Armas and Macía [20], the primary public healthcare centers were categorized into urban, semi-urban, semi-rural, and rural healthcare centers.

#### 2.3.2. Nurse Prescribing Self-Efficacy Scale (NP-SES) 

NP-SES was developed by Galiana–Camacho et al. [8] in 2021. The researchers designed a questionnaire of 19 items that were scored on a 100-point scale (0 = totally sure I cannot do it to 100 = totally sure I can do it) with a minimum score of 0 points and a maximum of 190 points. NP-SES has three dimensions: clinical assessment and pharmacological knowledge (items 1 to 5), supplementary prescribing and evidence-based practice (items 7 to 10), and independent prescribing and patient education (items 6 and 11 to 19). An average of all items is obtained to calculate total and dimensions scores, which are then converted to a 100-point scale, in order to facilitate the interpretation of nurses’ self-efficacy in NP. Internal consistency of the original scale was 0.958 for global composite score, 0.864 for clinical assessment and pharmacological knowledge, 0.914 for supplementary prescribing and evidence-based practice, and 0.951 for independent prescribing and patient education dimension [8]. G-NP-SES was the terminology used to refer to the Galician version (Supplementary Materials, Table S1).

The online questionnaires, administered through the Google Forms platform, were anonymous and self-completed between March and June of 2022. Participants were free to omit any questions they did not want to answer. No incentive was offered for completing the questionnaire.

### 2.4. Development and Clinical Validation of the Galician Version of the NP-SES (G-NP-SES) (Figure 1)

The translation and validation of the NP-SES for use in Galicia (Spain) were authorized by the author of the original instrument, Dr. Hernández-Padilla.

**Figure 1:**
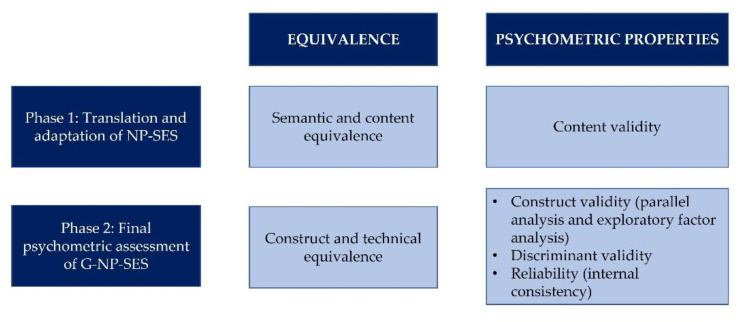
Procedure for establishing equivalence and psychometric properties of the G-NP-SES. Abbreviations: G-NP-SES. Galician version of the Nurse Prescribing Self-Efficacy Scale; NP-SES. Nurse Prescribing Self-Efficacy Scale.

#### 2.4.1. Development of the Galician Version of the NP-SES (G-NP-SES)

The cross-cultural adaptation process followed the steps outlined by Beaton et al. [21]. The method developed by Sperber et al. [22] was used for establishing semantic equivalence and validating the translated instrument. The translation and back-translation were completed by bilingual translators who were native speakers in the target language. Each item in the original and back-translated versions was ranked in terms of comparability of language and similarity of interpretability. Likert scales ranging from 1 (extremely comparable/extremely similar) to 7 (not at all comparable/not at all similar) were used for ranking by participants, who were fluent in English. Any mean score > 3 required a formal review of the translation. After several minor changes, the Galician translation was deemed to be semantically equivalent to the original version. 

A pilot study was conducted with 15 nurses to test the comprehensibility and legibility of the G-NP-SES. The comprehension of the questions was evaluated by respondent debriefings with two NP experts, who asked questions in a standardized interview setting immediately after the participants had filled in the questionnaires. The pilot participants reported ease in completing the questionnaire and a good level of comprehension was confirmed, so only minimal changes were made following the pilot study (Supplementary Materials, Table S1).

#### 2.4.2. Psychometric Assessment of the G-NP-SES

The G-NP-SES’ reliability and validity were assessed.

- Reliability
  We computed the scale’s Cronbach alpha (α), the corrected item–total correlation (C-ITC), and the scale’s α when the item was removed in order to test the tool’s internal consistency reliability. From the item–total statistics, items were considered for removal if any of the following two criteria were met: (1) the C-ITC was below 0.30; (2) the item caused a substantial drop (10% or more) in the scale’s α score when removed [23].
- Validity
  The G-NP-SES’ content validity was assessed by a panel of nurses. All the experts were active nurses with more than five years nursing experience. Experts used a 4-point Likert scale to evaluate the relevance of each item, from 1 being “not relevant at all”, to 4 being “highly relevant”. For each item, the content validity index (I-CVI) was calculated as the proportion of respondents answering 3 or 4 on the scale. Furthermore, the total scale content validity index (S-CVI), a mean of the I-CVI for all items, was calculated. A minimum cut-off I-CVI and S-CVI of 0.78 and 0.90, respectively, was used [24].
  The G-NP-SES’ construct validity was assessed by conducting an exploratory factor analysis (EFA). First, we computed the Kaiser–Meyer–Olkim measure (KMO) and the Bartlett’s sphericity test to determine the adequacy and suitability of the sample. A KMO higher than 0.70 and a significant Bartlett’s sphericity test (*p* < 0.05) were considered as evidence of the appropriateness to conduct an EFA [25]. Then, we ran an EFA using principal component analysis with Varimax rotation. The number of factors to consider was determined by Kaiser’s criteria (eigenvalue >  1 rule) and parallel analysis which utilized the rawpar.sps script developed by O’Connor (https://oconnor-psych.ok.ubc.ca/nfactors/rawpar.sps, accessed on 16 December 2022). A factor loading value equal to or higher than 0.45 was considered acceptable [25,26].
  The G-NP-SES’ discriminant validity was tested using the square root of average variance extracted (AVE) and Pearson’s correlation coefficient [27].

### 2.5. Ethical and Legal Considerations

This study was performed with the approval of the Bioethics Committee of the University of Santiago de Compostela and Health Area Management of Santiago de Compostela and Barbanza.

By email, we contacted the original author, Dr. Hernandez-Padilla, and obtained permission to use the NP-SES.

After explaining the procedure and objective of the investigation, we obtained the participants’ consent and explained that their participation was completely voluntary. Pursuant to the Declaration of Helsinki and the Data Protection Act (Organic Law 3/2018), data confidentiality was guaranteed at all times.

### 2.6. Statistical Analysis

The results are presented as the number and percentage, mean and standard deviation, or median and interquartile range. Numerical (Kolmogorov–Smirnov test; skewness; kurtosis; and the relationships between the mean, median, and mode) and visual (Q–Q plot) methods were used to test the normality of the data.

The software IBM SPSS 27.0 was used for the statistical processing of the data. A *p*-value of less than 0.05 was considered significant throughout the study.

## 3. Results

### 3.1. Description of Sample

A total of 193 valid responses with no missing values for scale scores were extracted from the 200 completed questionnaires. The descriptive statistics for the participants are shown in Table 1.

### 3.2. Construct Validity Results

**Figure 2:**
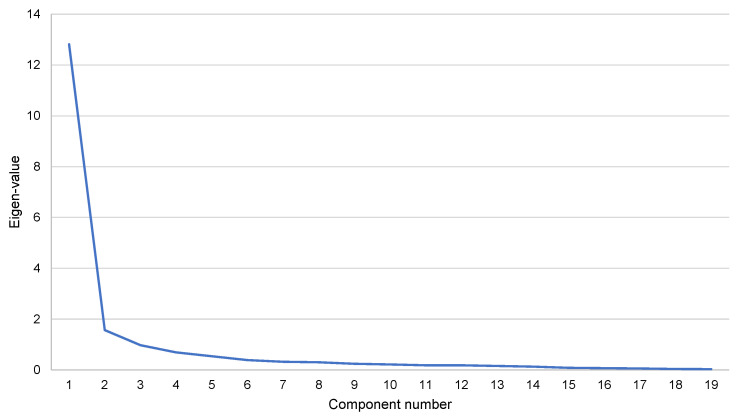
Scree plot of the eigenvalues of the factors for the Galician version of the Nurse Prescribing Self-Efficacy Scale (G-NP-SES). The scree plot shows that the eigenvalues of the first three dimensions are greater than 1.

The KMO test (KMO = 0.908), and the Bartlett’s sphericity test (ꭓ^2^(171) = 4842.19, *p* < 0.001) of the G-NP-SES showed common factors exist and are suitable for performing an EFA (Table 2). Principal component analysis was used to extract factors. Parallel analysis and eigenvalues suggested a model with three dimensions that accounted for 80.82% of the variance found. Three factors presented eigenvalues >1 (Figure 2) and were validated by parallel analysis (Table 3). Varimax rotation was used to improve factor interpretability and all the items loaded higher than >0.75 into the corresponding factor. 

### 3.3. Content Validity Results

The results showed that the G-NP-SES had the I-CVI ranging from 0.87 to 1 and the S-CVI at 0.92, indicating high content validity.

### 3.4. Reliability Results

Table 4 summarizes the internal consistency results, as well as the descriptive data, for all the G-NP-SES’ items. Correlation analysis showed that the scores of all items were significantly and positively correlated with the total score. The G-NP-SES’ α was 0.90 (Table 5), which would not have increased if any of the items had been removed. Furthermore, all the items’ C-ITCs were higher than 0.3. 

Table 5 shows the α of the G-NP-SES and its three factors, as well as the participants’ mean score, for the G-NP-SES and all its subdimensions.

### 3.5. Discriminant Validity Results

For testing discrimination between factors, the square root of AVE was checked for whether it is greater than the magnitude of the correlation between factors (Table 6). The square root of AVE ranged from 0.86 to 0.88 and correlation coefficients ranged from 0.58 to 0.62; thus, discriminant validity was established.

## 4. Discussion

In this study, the NP-SES was translated and culturally adapted into Galician for the first time, and it was verified among Galician nurses. This study provided scientific evidence for the application of the G-NP-SES to Galician nurses working in primary healthcare centers since it has good psychometric properties, consistent with that of the original Spanish version. Taking into account that NP has been a right recently granted to Galician nurses [28], this novel tool might be very useful in identifying gaps in nurses’ confidence to prescribe and tailor educational programs to build their self-efficacy.

Spain is a decentralized and plurilingual state with advanced language planning legislation. Although Spanish is the only official language across Spain, there are also established minority languages such as Galician, which is co-official with Spanish in Galicia, an autonomous region of Spain. The minority languages, in addition to being a means of communication, are as much a part of their speakers’ identities as any other cultural aspect, which encompass a wide range of values and beliefs [29]. The promotion of less widely used European languages represents an important contribution to multilingualism, which is regarded as an asset in terms of creativity and innovation, because people who speak more than one language are more adept at dealing with more divergent thinking, creativity, and the sensitivities of communicative [30].

At the beginning of the twenty-first century, Galician was classified by the UNESCO as an “endangered language”, as inter-generational transmission was failing [31]. However, nowadays it is no longer a language threatened with disappearance because of its inherently strong position and its close proximity to Portuguese — they descend from the same language [32]. The proximity to Portuguese is such that it is estimated that they have around 85% of their vocabularies in common, which allows communication between Galicians and people who live in countries where Portuguese, the fifth most spoken language in the world, is the official language (Portugal, Brazil, etc.) [33]. 

In Galicia, the nursing profession has recently experienced an expansion of competences since the regularization of NP [28]. This new situation demands that nurses deliver beyond their originally devised tasks and roles, so it is very interesting to assess factors that can facilitate a better adaptation to it. Among these factors, self-efficacy (a modifiable cognitive element tightly linked to workplace wellbeing) [9], and the ability to handle new challenges [34], stand out. This observation may be explained by the positive relationship that exists between self-efficacy and job satisfaction [35], more optimistic thoughts [36], effort expenditure towards carrying out an activity [37], and/or the level of motivation [37]. However, it is worth mentioning that it is necessary to measure self-efficacy in specific contexts instead of self-efficacy in general [8]. Thus, instruments designed specifically to assess nurses’ self-efficacy in NP are necessary.

The translation procedure and analysis of the data revealed good content validity, discriminant validity, and internal consistency of the scale. All the items’ I-CVI and the G-NP-SES S-CVI were excellent [24], and α reliability of the G-NP-SES and its dimensions fell into Devellis’s [38] “very good” range, indicating acceptable reliability. Although these results were similar to those obtained in the original Spanish version [8], α reliability of the NP-SES was slightly higher (0.958). In general, an α value higher than 0.9 suggests redundancy among the items [39], but it also supports that the NP-SES measures the construct of interest [40]. In fact, all the items comprising the NP-SES contributed to measuring self-efficacy of important aspects of the NP process [17]. 

The factor analysis method was used to simplify a matrix of correlations so the relationship between items in a scale could be more easily understood [41]. The results from the EFA revealed that the underlying structure of the G-NP-SES comprised well-defined and highly interpreted three factors, the same ones as those reported by Galiana –Camacho [8], and whose analysis allowed the assessment of nurses’ self-efficacy in all the actions involved in the prescribing process [17]. However, it is important to highlight the unidimensional structure suggested by the sharp point of inflection after the first eigenvalue of the scree plot (Figure 2), which would also be supported by the high ratio (>4:1) between the first and second eigenvalues [42]. Again, these data reaffirm the idea that all the items of the G-NP-SES assess the self-efficacy in NP [8].

Several limitations of the study must be mentioned. First, as participants filled in questionnaires themselves, there may be some self-report bias. Participants might answer according to researchers’ expectations. Second, the study included nurses working in primarily public healthcare centers, which may limit the external validity of our findings. Because of this limitation, additional studies are needed to determine if the results from our study can be generalized to nurses who work in hospital settings or in the private sector in Galicia. Third, the questionnaire was available in Spanish and Galician only. Its translation into English and its validation could be beneficial to, and implemented by, many more nurses worldwide.

## 5. Conclusions

G-NP-SES was validated as a reliable assessment tool to evaluate the Galician nurses’ self-efficacy to prescribe and consequently to improve their job performance. Using G-NP-SES, it is possible to formulate programs for improving the level of confidence that nurses have for all the main actions of the NP process.

## Figures and Tables

**Table 1:** Sociodemographic characteristics of the study’s participants (*n* = 193).

	All Participants n (%)
Sex, n (%)	
Male	28 (14.3)
Female	165 (85.7)
Age, median (IQR)	32 (25–37)
Primary care center, n (%)	
Urban	90 (46.6)
Semi-urban	68 (35.2)
Semi-rural	22 (11.4)
Rural	13 (6.7)
Years of experience, n (%)	
0–5 years	90 (46.6)
6–10 years	30 (15.5)
11–15 years	30 (15.5)
16–20 years	18 (9.3)
>20 years	25 (13)
Education, n (%)	
Nursing degree before implementation of Bologna Process ^a^	85 (44)
Nursing degree after implementation of Bologna Process	108 (56)
Specialist nurse	38 (19.7)
Postgraduate or doctorate	19 (5.2)

^a^ The Bologna Process is a series of ministerial meetings and agreements between European countries to ensure comparability in the standards and quality of higher-education qualifications. Abbreviations. IQR. interquartile range.

**Table 2:** Exploratory factor analysis (EFA) results and structure of the Galician version of the Nurse Prescribing Self-Efficacy Scale (G-NP-SES) (*n* = 193).

	Factor 1	Factor 2	Factor 3
**Item 1.** Perform an effective initial clinical assessment regardless of patients’ health situation	**0.861**	0.303	0.356
**Item 2.** Identify all available treatment options to improve patients’ health problems regardless of their complexity	**0.907**	0.344	0.272
**Item 3.** Give appropriate administration instructions for any medicine that I prescribe	**0.889**	0.291	0.231
**Item 4.** Consider the interactions or contraindications of drugs regardless of patients’ health situation	**0.895**	0.329	0.157
**Item 5.** Identify the side effects or adverse reactions of prescribed drugs regardless of patients’ health situation	**0.845**	0.273	0.287
**Item 6.** Treat any adverse or allergic reaction due to prescribed medicines, dressings, or other products	0.196	0.226	**0.865**
**Item 7.** Search for up-to-date evidence before prescribing medicines, dressings, or other products at all times	0.317	**0.912**	0.261
**Item 8.** Access up-to-date protocols and clinical guidelines created by local authorities in prescribing to inform my decisions	0.241	**0.865**	0.338
**Item 9.** Follow the protocols and clinical guidelines that are appropriate for each individual patient according to local authorities’ recommendations	0.268	**0.823**	0.165
**Item 10.** Make decisions regarding the prescribing process in conjunction with other health care professionals	0.154	**0.817**	0.245
**Item 11.** Differentiate situations in which I can prescribe independently from those in which I need to follow existing protocols	0.212	0.240	**0.793**
**Item 12.** Decide when it is appropriate to initiate a new treatment for patients regardless of the situation	0.206	0.211	**0.901**
**Item 13.** Decide when it is appropriate for patients to continue with their treatment regardless of the situation	0.255	0.229	**0.955**
**Item 14.** Decide when it is appropriate to modify patients’ treatment regardless of the situation	0.112	0.219	**0.921**
**Item 15.** Decide when it is appropriate to cease patients’ treatment regardless of the situation	0.248	0.143	**0.921**
**Item 16.** Educate patients on the therapeutic use and the risk of interactions for any medicine, dressing, or other product that I prescribe	0.110	0.115	**0.807**
**Item 17.** Educate patients on how to look for side effects of any medicine, dressing, or other product that I prescribe	0.204	0.317	**0.859**
**Item 18.** Evaluate the effects that the education provided on prescribed items has on patients	0.334	0.142	**0.884**
**Item 19.** Evaluate patients’ adherence to treatment after prescribing them medicines, dressings, or any other products	0.334	0.239	**0.895**
**% of variance**	67.44	8.26	5.12
**% of cumulative variance**	67.44	75.7	80.82

Bold font indicates the items that comprise the factors. The three factors represent the following dimensions of nurse prescribing: Factor 1 = clinical assessment and pharmacological knowledge; Factor 2 = supplementary prescribing and evidence-based practice; Factor 3 = independent prescribing and patient education [8]. The name of the items can be found in Table 2 and Table S1.

**Table 3:** Results of parallel analysis.

Factor	Original Eigenvalue	Mean of Random Eigenvalues	95th Percentile of Random Eigenvalues
1	12.812749	1.603668	1.718583
2	1.569727	1.381483	1.460722
3	1.193070	1.104340	1.158626
4	0.889622	1.016968	1.080745

Values of factors from the raw data (column 1) which exceed the 95th percentile values (column 3) can be considered actual factors.

**Table 4:** Internal consistency results (*n* = 193).

	C-ITC	Alpha (α) if Item Deleted	Mean (SD)
Item 1	0.828	0.891	61.76 (22.75)
Item 2	0.727	0.892	56.48 (24.60)
Item 3	0.821	0.891	64.30 (23.51)
Item 4	0.897	0.890	58.39 (23.94)
Item 5	0.914	0.890	58.03 (25.44)
Item 6	0.782	0.891	59.69 (23.80)
Item 7	0.813	0.8971	68.08 (24.79)
Item 8	0.755	0.892	66.89 (23.93)
Item 9	0.717	0.892	69.48 (20.48)
Item 10	0.706	0.892	72.54 (19.51)
Item 11	0.815	0.891	64.15 (24.14)
Item 12	0.826	0.891	55.70 (24.21)
Item 13	0.840	0.891	58.76 (23.92)
Item 14	0.806	0.891	56.79 (23.52)
Item 15	0.710	0.892	58.5 (23.50)
Item 16	0.773	0.892	66.17 (21)
Item 17	0.804	0.891	67 (20.7)
Item 18	0.844	0.891	65.39 (21.16)
Item 19	0.767	0.892	71.35 (20.85)

Abbreviations. C-ITC. Corrected Item-Total Correlation; SD. Standard deviation.

**Table 5:** Chronbach’s alpha (α) and descriptive data for the G-NP-SES and its subdimensions.

	Chronbach’s Alpha (α)	Total Sample Mean (SD)
G-NP-SES	0.90	63.12 (18.83)
Factor 1	0.88	59.79 (22.07)
Factor 2	0.86	66.04 (20.19)
Factor 3	0.89	63.63 (18.57)

Factor 1 comprises item 1 to 5, factor 2 comprises item 7 to 10, and factor 3 comprises item 6 and item 11 to 19. The three factors represent the following dimensions of nurse prescribing: factor 1 = clinical assessment and pharmacological knowledge; factor 2 = supplementary prescribing and evidence-based practice, and factor 3 = independent prescribing and patient education [8]. The name of the items can be found in Table 2 and Table S1. Abbreviations. G-NP-SES. Galician version of the Nurse Prescribing Self-Efficacy Scale; SD. Standard deviation.

**Table 6:** Correlation matrix among factors of G-NP-SES.

	Factor 1	Factor 2	Factor 3
Factor 1	0.88		
Factor 2	0.616	0.855	
Factor 3	0.620	0.579	0.88

Diagonals represent the square root of the AVE. Factor 1 comprises item 1 to 5, factor 2 comprises item 7 to 10 and factor 3 comprises item 6 and item 11 to 19. The three factors represent the following dimensions of nurse prescribing: Factor 1 = clinical assessment and pharmacological knowledge; Factor 2 = supplementary prescribing and evidence-based practice; Factor 3 = independent prescribing and patient education [8]. The name of the items can be found in Table 2 and Table S1. Abbreviations: AVE. Average Variance Extracted.

## Data Availability

The data presented in this study are available on request from the corresponding author (S.N.) upon reasonable request. The data are not publicly available due to restrictions e.g., the study information could compromise the privacy of participants.

## Supplementary Materials

The following supporting information can be downloaded at: https://www.mdpi.com/article/10.3390/healthcare10122563/s1, Table S1: Galician version of the Nurse Prescribing Self-Efficacy Scale (G-NP-SES).
