# Supplementary material for: Translation, Cross-Cultural Adaptation, and Validation of the Galician Version of the Nurse Prescribing Self-Efficacy Scale
Source: Healthcare (Basel). 2022 Dec 17;10(12):2563. doi: 10.3390/healthcare10122563 (PMC9778594; doi:10.3390/healthcare10122563)
Supplement: Supplementary file 1 [file healthcare-10-02563-s001.zip › healthcare-2072587-supplementary.pdf]

Table S1. Galician version of the Nurse Prescribing Self-Efficacy Scale (G-NP-SES)

Indique o seu nivel de confianza coas afirmacións detalladas a continuación, empregando a seguinte escala:

0%	10	20	30	40	50%	60	70	80	90	100%
Totalmente seguro de que NON podo facelo					Moderadamente seguro de que podo facelo					Totalmente seguro de que SI podo facelo

**Á hora de usar ou indicar medicamentos ou produtos sanitarios estou seguro de que SEMPRE podo...**	% de confianza (0-100)
1. Realizar unha valoración inicial axeitada do paciente que necesita prescrición, independentemente do cadro clínico que presente.	
2. Identificar todas as opcións terapéuticas dispoñibles segundo o problema de saúde que presenta o paciente, independentemente da complexidade do problema.	
3. Dar as instrucións de administración axeitadas para calquera medicamento que receite.	
4. Valorar as contraindicacións da medicación ou produtos prescritos, independentemente de cal sexa a situación de saúde do paciente.	
5. Detectar os efectos secundarios ou reaccións adversas da medicación prescrita, independentemente de cal sexa a situación de saúde do paciente.	
6. Tratar unha reacción adversa non previsible ou reacción alérxica derivada da administración do medicamento ou produto sanitario prescrito.	
7. Consultar información actualizada baseada na evidencia sobre medicación ou produtos sanitarios, en todos os casos.	
8. Acceder a protocolos actualizados e guías de práctica clínica establecidos polas autoridades competentes en cada caso.	
9. Seguir os protocolos e guías de práctica clínica de forma individualizada, segundo as recomendacións da autoridade competente.	
10. Tomar decisións de forma conxunta con profesionais do equipo con respecto ao proceso de prescrición.	
11. Diferenciar situacións nas que podo prescribir de forma independente de aquelas nas que necesito seguir os protocolos existentes.	
12. Decidir cando é apropiado iniciar un novo tratamento dos pacientes independentemente da situación.	
13. Decidir cando é apropiado que os pacientes continúen co seu tratamento independentemente da situación.	
14. Decidir cando é apropiado modificar o tratamento dos pacientes independentemente da situación.	
15. Decidir cando é axeitado cesar o tratamento do paciente independentemente da situación.	
16. Educar aos pacientes sobre o uso terapéutico e o risco de interaccións de calquera medicación, apósito ou outro produto que prescriba.	

17.	Educar aos pacientes sobre como buscar efectos secundarios de calquera medicamento, apósito ou outro produto que receite.
18.	Avaliar os efectos que teñen nos pacientes a educación proporcionada sobre os medicamentos/produtos prescritos.
19.	Avaliar a adherencia dos pacientes ao tratamento despois de receitarlles medicamentos, apósitos ou calquera outro produto.
